# COVID-19 and the call for ‘Safe Hands’: Challenges facing the under-resourced municipalities that lack potable water access - A case study of Chitungwiza municipality, Zimbabwe

**DOI:** 10.1016/j.wroa.2020.100074

**Published:** 2020-10-16

**Authors:** Luckson Zvobgo, Pierre Do

**Affiliations:** a Climate System Analysis Group (CSAG), University of Cape Town, Rondebosch, 7701, Cape Town, South Africa; b Department of Hydraulic Engineering, State Key Laboratory of Hydroscience and Engineering, Tsinghua University, Beijing, 100084, China

**Keywords:** COVID-19, Safe hands, Intermittent water supply, World health organisation, Chitungwiza

## Abstract

Billions of people living in developing countries lack access to safe drinking water, not to mention water for handwashing, one of the most effective ways to contain the fast spreading novel coronavirus (COVID -19). The recent global spread of COVID-19 has fostered diverse initiatives such as the ‘Safe Hands’ challenge led by the World Health Organization. Individuals are encouraged to regularly wash their hands for 40–60 s under running water with soap. This call for ‘Safe Hands’ comes at a time when water insecurity and limited access to handwashing facilities in Africa is heightened. In this article, Chitungwiza city in Zimbabwe is used as a case study to assess the implications of the ‘Safe Hands’ challenge for poor municipalities in developing countries and characterize the challenges they face. To do so, interviews were conducted at water points/boreholes used by residents during Zimbabwe’s COVID-19 national lockdown. The calculation of water requirements for proper hand hygiene determined the capacity for water-stressed regions to effectively implement ‘Safe Hands’. On average, it was established that one person consumes an extra 4.5 L per day of water when they practice WHO ‘Safe Hands’ in the context of COVID-19. This increases domestic water demand in Chitungwiza by 9%. Due to water scarcity, people in Chitungwiza were experiencing challenges with practicing ‘Safe Hands’. With their ‘dry taps’ woes, they might not be able to meet the standards of this WHO challenge. Lack of soap also reduced the effectiveness of the ‘Safe Hands’ challenge. This paper proposes short- and long-term measures that would allow effective implementation of the ‘Safe Hands’ by means of sustainable potable water supply. These measures include extensive social awareness and temporary change of household water use behavior. Municipalities are recommended to establish public private partnerships (PPPs) to create immediate and long-term water investments. Structural and transformational reforms would enhance, through flexible planning, investments for both water infrastructure and governance. This narrative has the potential to improve the urban water systems resiliency against future pandemics.

## Highlights

• Some suburbs had 4 months of dry taps despite water being a vital weapon to prevent COVID-19.
• Households resort to using less water due to lack storage facilities & long queues at boreholes.
• Residents could not obey the lockdown regulation as they queue at water points in crowds.
• Lack of soap at households affect the implementation of safe hands & its effectiveness.
• Lack of access to water is among the challenges people in Chitungwiza are facing to fight COVID-19.

## 1 Introduction

On March 22, 2020, the world celebrated World Water Day with grief and anguish while undergoing the COVID-19 pandemic (the illness caused by SARS-CoV-2) affecting millions and killing hundreds of thousands of people. The preceding week on March 13, the World Health Organisation (WHO) introduced the ‘Safe Hands’ challenge. The ‘Safe Hands’ encourages individuals across the globe to regularly wash their hands for 40–60 s with soap under running water to maintain hygiene^1^ and limit the virus transmission. The underlying rationale is clear: washing hands regularly and thoroughly physically degrades and removes viral particles (Staddon et al., 2020), therefore, it lowers the likelihood of infection transmission. While millions of people living in developing countries lack access to potable water, this affect the implementation of handwashing activities, despite at this stage, handwashing being one of the most effective ways to contain the fast spreading coronavirus.

The increased potable water demand as a result of the ‘Safe Hands’ initiative is adding to the challenges that the municipalities in developing countries already face. Water supply in these regions often suffers from diverse issues: numerous and institutional bottlenecks, inadequate financial resources, rapid population growth (Ainuson, 2010; Zimmerman et al., 2008) and poorly maintained and aging infrastructure (Britto et al., 2019; Larsen et al., 2016). These problems prevent municipalities from providing reliable potable water to people, thus increasing the vulnerability of millions to diseases and pandemics such as COVID-19. This article will explore the implications of insufficient and unreliable water supplies on practicing ‘Safe Hands’ to prevent COVID-19 transmission for people in developing regions.

This article is also addressing competing water use arise between the increased potable water demand brought by COVID-19 through regular hand washing and the existing water rationing that under-resourced municipalities in developing countries implement to maintain sustainable water supply and management of water consumption. Short- and long-term measures are needed to improve water supply and access and create resilient water systems that mitigate the shocks of future disasters and pandemic diseases. This is essential to protect millions of people that live in the informal and densely populated areas within cities. As COVID-19 continues to spread to developing countries without sustainable access to safe drinking water, people in these regions remain highly vulnerable (Aboelnga et al., 2020). There is a strong agreement that people in developing regions lack access to reliable water supply and hand washing facilities (World Health Organisation, 2020).

Globally, 2.1 billion people lack access to clean and safe water, they are using either an unimproved water source or an improved source that is contaminated with faecal matter (World Health Organisation, 2016). According to UNICEF and WHO (2019), as many as one in three of the world’s people do not enjoy access to safe and reliable water services. Three billion people across the world do not have basic hand-washing facilities (soap and water) in their home. Increasing water scarcity in urban areas is projected to intensify even more during the coming years. Almost 900 million people live in cities that are facing seasonal water shortages, in which potable water supplies dip below 100 l/day at least one month each year. By 2050, these numbers are expected to grow to 1 billion and 3.1 billion people, respectively (Britto et al., 2019). Nearly a third of humanity, including over half of the 6 billion urban dwellers predicted by 2050, will face critical water shortages in their cities (Xylem, 2013). In Africa, 400 million people lack access to clean water.^2^ African cities are also facing increasing water insecurity (Grasham et al., 2019). The COVID-19 pandemic exacerbates this situation. In such circumstances, planning and governance responses need to be both flexible and proactive in order to manage this shock and its associated effects (Rodina, 2019).

Access to clean water including handwashing facilities, is widely accepted as a cost-effective way to reduce the disease burden in lower income countries (Jamison, 2018). With evidence provided in literature, household water insecurity may exacerbate the COVID-19 pandemic, especially in Africa, Asia and Latin America, as many people do not have access to safe and secure potable water services (Staddon et al., 2020). The problem is exacerbated by the continuous development of disordered informal network, adding to the alarming state of current affairs. About 238 million people in sub-Saharan Africa (SSA) are living in informal settlements without basic services i.e., access to water to practice basic hygiene, and social amenities.^3^ They can, therefore, hardly comply with the COVID-19 transmission-prevention measures. This places millions of people at risk of the fast spreading COVID-19 in SSA.

Water provision in developing regions is mainly provided through municipalities. These municipalities are often under-resourced (Jacobsen et al., 2013) and are exposed to severe water supply rationing. Urban water scarcity in developing countries is getting more real, compelling, and growing day by day (Britto et al., 2019). The outbreak of COVID-19 intensifies this situation as people are required to frequently do handwashing. The frequent handwashing activities require household taps to always have running water. The ‘Safe Hands’ challenge encounters the existing water insecurity problems and its implementation faces hurdles. This has the potential to reduce its effectiveness.

This study assesses the capacity to implement ‘Safe Hands’ for cities like Chitungwiza, Zimbabwe where residents have dry taps for almost 5 days every week.

## 2 Methods

### 2.1 Study area

**Fig. 1:**
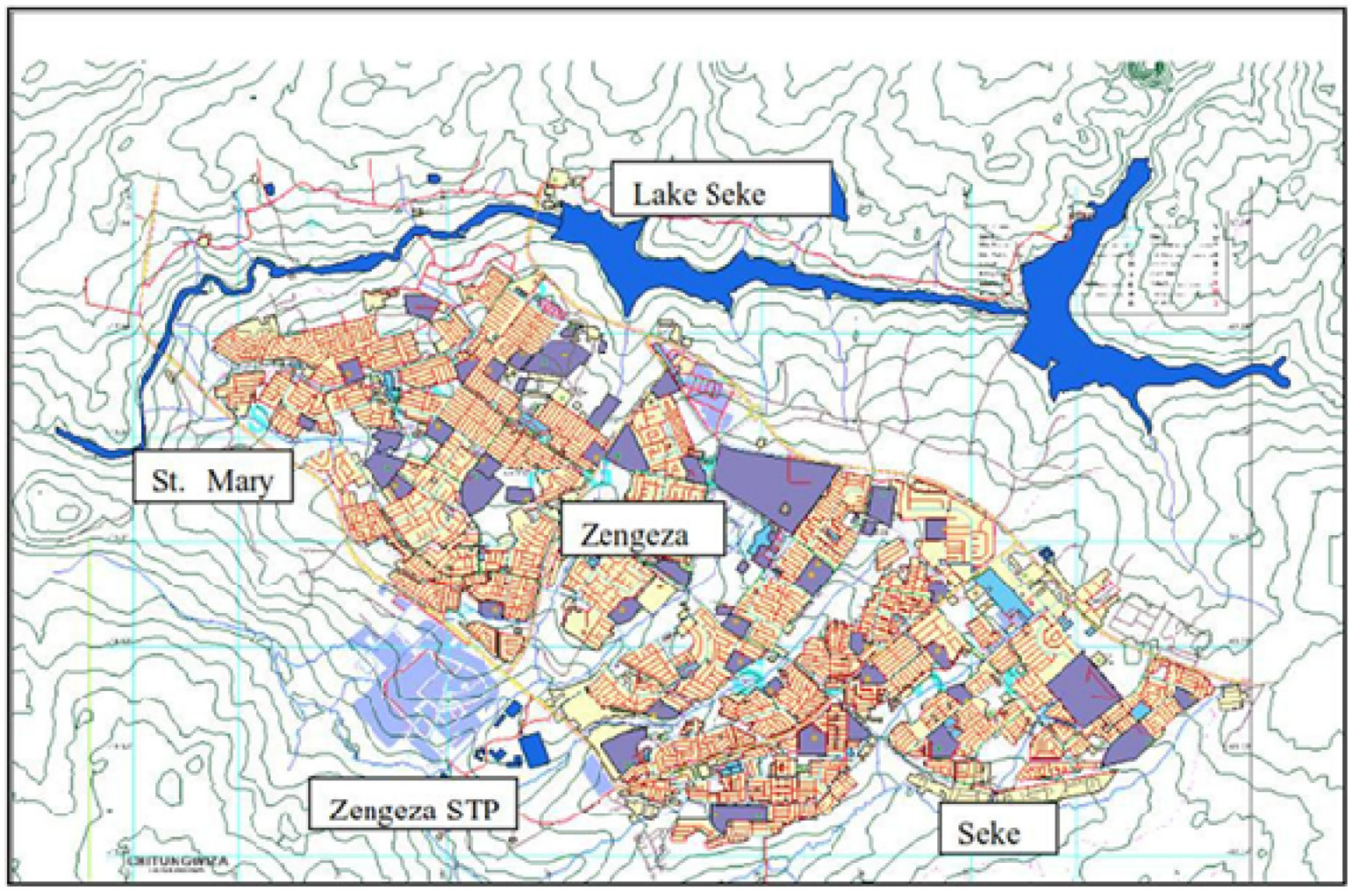
Map of the administrative boundaries of Chitungwiza urban and the four residential zones. **Source:** Japanese International Cooperation Agency (JICA) Report of 2013. A project for the improvement of water supply, sewage and solid waste management in Chitungwiza in the Republic of Zimbabwe.

Chitungwiza, Zimbabwe is used as a case study to highlight the implications and successes of the ‘Safe Hands’ challenge in under-resourced and poorly managed municipalities in developing countries. Chitungwiza is the third largest and fastest growing city in Zimbabwe with a population of over 386 000 people.^4^ The local municipality is responsible for supplying potable water. Years of economic meltdown and under-development in Zimbabwe have resulted in municipalities failing to keep up with supplying reliably to its residents (Japan International Cooperation Agency, 2013). Chitungwiza is a classic example of a cities failing to provide reliable water service. The municipality for the past decade has been implementing a strict water rationing program (Fig. 1).

### 2.2 Water supply in chitungwiza

Potable water in Chitungwiza is supplied through two sources: a potable water distribution system and municipal boreholes. The potable water system is supported by 79 municipal boreholes. Chitungwiza does not have its own potable water treatment plant; rather, it buys potable water from Harare Water. Therefore, for Chitungwiza to sustainably maintain its supply, residents must pay for water services so that the municipality is able to buy water from City of Harare. Domestic water supply in Chitungwiza is divided into four zones. A total of 26 suburbs are in the four zones.^5^ The water connections statistics in the four residential zones are given in Table 1.

**Table 1:** Metered domestic water connections in Chitungwiza.

Zone	Suburbs/Units	Number of metered connections
**Seke North**	Unit A, B, C, E, F, H, N, O & P	15 572
**Seke South**	Unit M, L, J, K and D	13 620
**Zengeza**	1, 2, 3, 4 and 5	16 165
**St Mary’s**	St Mary’s’, Manyame Park Phase 1- 5	10 067

Chitungwiza municipality receives approximately 28–30 ML of water per day from Harare Water against a daily demand of approximately 45 ML (Gambe, 2019). This has caused a water supply deficit leading to a strict water rationing in Chitungwiza. Each zone only has access to water services for 36–48 h per week. More than 40% of the residential suburbs in Chitungwiza experience deficiencies in water supply lasting from 24 h to several days (Africa Water Facility, 2009). The current supply volume from Harare was reduced because Chitungwiza municipality is owing Harare Water. The water supply volume from Harare Water is based on the purchasing power of the Chitungwiza municipality. The current intermittent water supply situation facing Chitungwiza, together with COVID-19, threatens people as they need a reliable and constant supply of water to safeguard public health. Immediate measures are required to address this intermittent water supply situation in Chitungwiza to curb the spread of COVID-19, along with long-term sustainable solutions to build resilience to future pandemics.

#### 2.2.1 Chitungwiza water distribution system

**Fig. 2:**
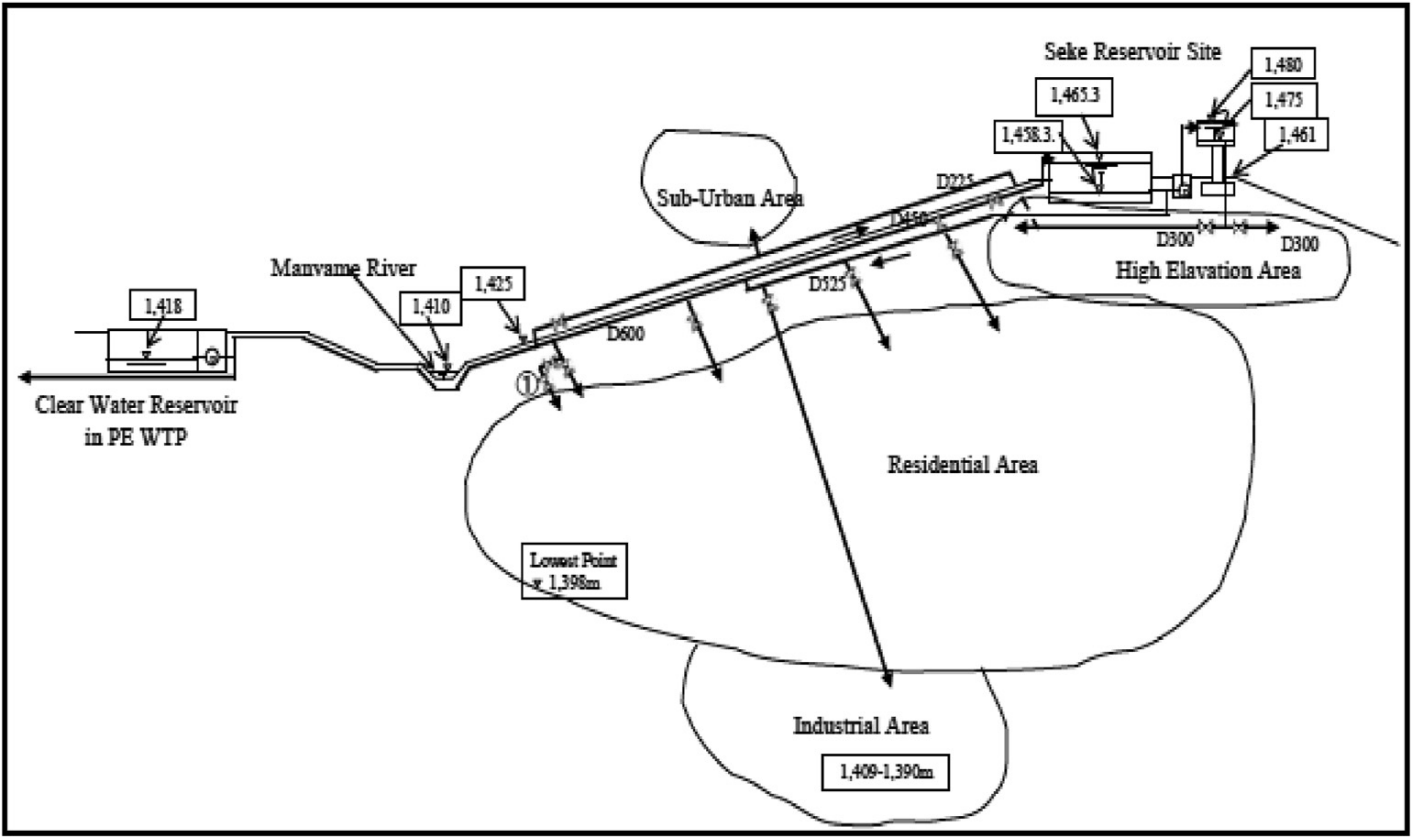
Water supply distribution system for Chitungwiza. **Source:** Japanese International Cooperation Agency (JICA) Report of 2013. A project for the improvement of water supply, sewage and solid waste management in Chitungwiza in the Republic of Zimbabwe.

The main water distribution pipeline that supplies water to Chitungwiza comes from the Prince Edward Water Treatment Plant. It leaves the treatment plant at an elevation of 1467 m in order to get to Chitungwiza. At this elevation, lower altitude areas of Chitungwiza (Fig. 2), Zengeza and St Mary’s’ zones, are fed directly using gravitational force. This main distribution pipeline is 600 mm in diameter and it splits into two mains of 525 mm and 450 mm to the night storage reservoir (NSR) in the Seke north zone (Fig. 2). The NSR supplies the two Seke zones (Seke north and south) because they are located in higher elevated ground that requires pumping from the NSR to boost water pressure. The initial NSR design storage capacity was 44 ML for three days. Due to population growth and city expansion, the NSR is now unable to supply water for at least two days to these two zones. This current situation has caused irregularities in water supply across the four zones. The problem is exacerbated by a lack of meaningful infrastructure development in the water system for the past three decades.

### 2.3 ‘Safe hands’ challenge

WHO made a call for policy makers to provide the necessary infrastructure for effectively implementing the ‘Safe Hands’ challenge in private and public places. This news has been unfortunately received with contempt in towns and cities in developing countries as most lack access to basic water services. Box 1 details the WHO ‘safe hands’ procedure that is relevant for this paper.

Box 1 **WHO ‘safe hands’ challenge procedure for water and soap method.**

In a video statement, the WHO Director General said there are several practical measures you can take to protect yourself from the new coronavirus. One of the most important is regular, safe and effective hand hygiene using soap and water. Here are the steps recommended by WHO.

1) Wet hands with water
2) Apply enough soap to cover all hands surface
3) Rub hands palm to palm
4) Right palm over left hand, interfaced fingers and vice versa
5) Palm to palm, finger interplaced
6) Back of fingers to opposing palms fingers interlocked
7) Rotational rubbing of left and right thumbs
8) Rotational rubbing with clasped fingers
9) Rinse hands with water
10) Dry thoroughly with a towel
11) Use towel to turn off the tap

Now I am calling on the world to take the WHO Safe Hands Challenge to be ready for coronavirus.

Source: World Health Organisation, The Safe Hands Challenge, https://www.youtube.com/watch?v=y7e8nM0JAz0.

Alt-text: Box 1

In a video statement, the WHO Director General said there are several practical measures you can take to protect yourself from the new coronavirus. One of the most important is regular, safe and effective hand hygiene using soap and water. Here are the steps recommended by WHO.

The procedure in Box 1 is possible when individuals have access to adequate water services. Using the procedure in Box 1, we determined how many litres people can use per day when they practice ‘safe hands’ in the context of COVID-19. Many health agencies are recommending washing hands for a minimum of 20 s up to 8–10 times per day (Staddon et al., 2020). Box 2 highlights the key message on when people should wash their hands as recommended by UNICEF and WHO during the COVID-19 pandemic.

Box 2 **When to practice ‘safe hands’ in the context of COVID-19.**

When should I wash my hands?

In the context of COVID-19 prevention, you should make sure to wash your hands at the following times:

• After blowing your nose, coughing or sneezing
• After visiting a public space, including public transportation, markets and places of worship
• After touching surfaces outside of the home, including money
• Before, during and after caring for a sick person
• Before and after eating

Source: UNICEF https://www.unicef.org/coronavirus/everything-you-need-know-about-washing-your-hands-protect-against-coronavirus-covid-19.

Alt-text: Box 2

Using Box 2, we assumed that a person would practice safe hands three times a day during the COVID-19 pandemic in the context of developing regions where water access is very limited.

### 2.4 Implications for daily water demand

We calculated the key aspects below of water supply service provision related to daily water consumption using recommended benchmarks and standards of best practices and the WHO (2010) guidelines:

a) Daily water consumption during the 40–60 s of handwashing: the key Assumption is that one person will practice ‘Safe Hands’ with running water on average three times a day to keep maintain good hygiene.
b) Daily increase of water consumption: the increasing water use per person per day and the implications of this new demand to the existing Chitungwiza water supply difficulties were determined.
c) Shift in water management practices: while the municipality does not provide sufficient amount of water before and during the COVID-19 pandemic, new water use practices and management were recommended to the municipalities and water users in the context of ‘Safe Hands’ challenge.

Using WHO (2010) guidelines for the minimum amount of water sufficient per person per day, 50 L were allocated per person per day. Box 3 highlights the summary of the results on hypothetical old and new demand for Chitungwiza in its simplistic form. Despite high non-revenue water in Chitungwiza, like in many cities in developing countries (Rouse, 2014), the research did not address water losses through leakages. The water demand increase referred to is specifically related to water discharge at household taps/standpipes.

### 2.5 Interviews

To assess the implications of the lack of water access for residents to practice ‘safe hands’ challenge in Chitungwiza, in-depth interviews were conducted at selected municipal (public) boreholes/water points. Four boreholes were visited in the four residential zones of Chitungwiza. Participants interviewed were local people collecting water, as their household taps were dry (https://www.youtube.com/watch?v=yzNwewfhzi0). Twelve in-depth interviews, three from each water point were conducted. The interviews were conducted on the 6th day of Zimbabwe’s national COVID-19 lockdown and 26 days after WHO initiated the ‘Safe Hands’ challenge. Interviews were conducted by Bustop TV, a local private media entity. It is important to note that the authors utilised the interviews as secondary users.

The interviewers solicit the views of the residents on the current water supply situation and the threats posed by water shortages for spreading of COVID-19 in Chitungwiza. The views expressed during these interviews were used to examine how best to address the intermittent water supply challenge in Chitungwiza in regard to the current COVID-19 pandemic and future outbreaks. The argument was also based on the knowledge of the authors on the current water supply challenges in Chitungwiza.

Video showing interviews, found on this link https://www.youtube.com/watch?v=yzNwewfhzi0.

## 3 Results and discussion

### 3.1 Water demand and consumption as a result of WHO ‘safe hands’ challenge

The study established that the implementation of ‘Safe Hands’ increases water consumption by 4.5 L per person per day (see Box 3). The results assume that one-person practice safe hands on average three times a day for 40–60 s. This is a 9% increase in the current domestic water consumption for Chitungwiza. Our results agree with Staddon et al. (2020); the authors found that if hands are washed in running water in the context of COVID-19, the average hand basin tap uses 2–3 L per minute, which implies a total water requirement of 8–10 L of clean water per person per day, if one practices ‘safe hands’ for 8–10 times as well as appropriate soap and drying facilities. Box 3 summaries the findings for the calculated the new demand and the amount of water required for safe hands if practiced for 40–60 s.

Box 3 **Results on the new demand for practicing ‘safe hands’ challenge.**

Old demand

(1) $$\begin{array}{l} \text{Current}\;\text{consumption}\;=\;\text{Population}\;\text{x}\;\text{Water}\;\text{use}\;\text{per}\;\text{person}\;\text{per}\;\text{day}\\ \;\;\;\;386\;000\;\text{x}\;50\text{l}\;=\;19\;300\;000\;\text{litres} \end{array}$$

19.3 ML (mega litres) hypothetically required in Chitungwiza for domestic use per day.

Using best standards practices, WHO guidelines and repeated demonstrations, we established that one person use 1.5**l** of water in 40–60 s of safely and thoroughly washing hands. Staddon et al. (2020) found that if a person’s hands are washed in running water, the average hand basin tap uses 2–3 L per minute, which implies a total water requirement of 8–10 L of clean water per person per day assuming they practicing safe hands for 8–10 times.

Assumption: Based on the notes provided in Box 1, Box 2, we assumed that one person practices the ‘safe hands’ challenge at least three times a day (for illustration purpose). Therefore, a person per day uses a total of 4.5 L of water washing hands. We added this amount to the current demand. The new water demand was calculated as follows:

Safe hands water consumption (SHWC)

(2) $$\begin{array}{l} \text{SHWC}\;=\;\text{Population}\;\text{×}\;\text{Water}\;\text{user}\;\text{per}\;\text{day}\;\text{for}\;\text{practicing}\;\text{safe}\;\text{hands}\\ \;=\;386\;000\;\times \;4.5\text{l}\;=\;1\;737\;000\;\text{litres} \end{array}$$

New demand

New Demand = Population x Water user per day for practicing safe hands + Existing water demand.

The total domestic water consumption for Chitungwiza increases by 9%. A 9% increment must be catered on a daily basis for achieving safe hands until the virus is contained. Thus;

(3) 19300000+1737000l=21037000litres

Alt-text: Box 3

### 3.2 Water supply situation in Chitungwiza: Insights from water point interviews

**Fig. 3:**
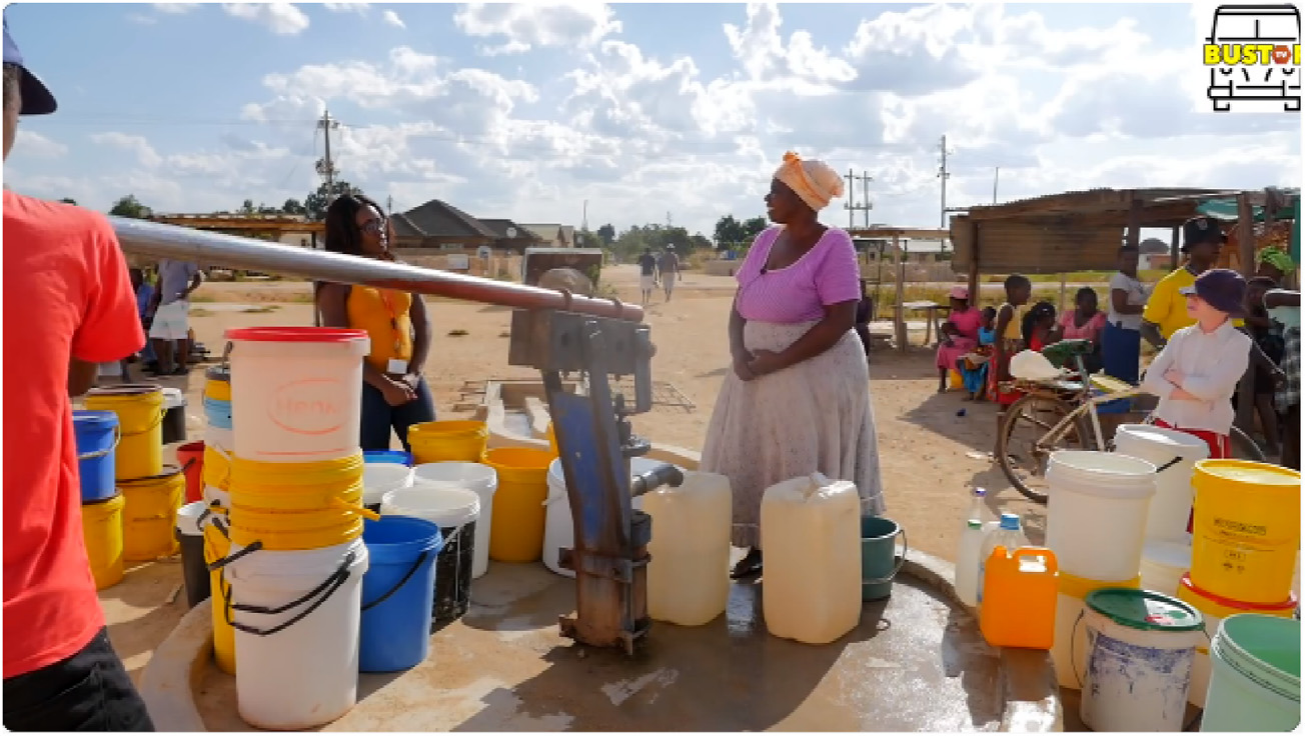
Long queues at a water point/borehole in Seke North zone, Chitungwiza. **Source:** Bustop TV interview.

Like the rest of the world, the Government of Zimbabwe (GoZ) declared 21 days of national lockdown to encourage the public to maintain social distancing. The aim of staying at home was to limit the spread of COVID-19. Despite the declaration, interviews revealed that due to the unavailability of water in Chitungwiza, people were gathering at municipal boreholes in long queues of more than 100 people (Fig. 3).

*“As you can see, we are over 100 here. No social distancing, we all want to collect water and go back home so we squeeze each other to get water”- Interviewee in Zengeza zone.*

The results confirm Staddon et al. (2020) reiterations that in many settlements in low-and-middle-income countries, insufficient water supply points lead to queuing for water and therefore present an extra challenge for social distancing, and thus too for preventing the spread of infectious diseases.

Many of the people queueing at boreholes were women and children, thus, increasing their vulnerability to COVID-19. Residents could not afford to stay home as they need to constantly fetch water for daily use and for practicing ‘Safe Hands’. The necessity of travelling to fetch water from water points makes it very hard to practice social distancing or self-isolation in Chitungwiza. Interviews further showed that residents in Chitungwiza understood the importance of lockdown and social distancing measures, but conditions such as the unavailability of potable water restrained their compliance, as they need water to frequently practice hand and surface-washing.

*“Lockdown is good, but the unavailability of municipal water forces us to go out to the boreholes to collect water. Yes, we have understood why we should stay home maintaining social distance. But, if the municipality could give us water twice a week, we can fill our containers at home, and less people can congest at boreholes during this COVID-19 lockdown” – Old woman at Zengeza water point.*

The frequency of going to water points/boreholes was intensified by residents’ lack of water storage facilities. This forced them to collect water daily, thus, increasing their risk of contracting COVID-19.

*“We come here daily. No council water at our houses. Our containers are few. We are afraid of COVID-19 but with this water situation we have no option. We have to come here to collect water daily” – Woman at St’ Marys’ water point*

**Fig. 4:**
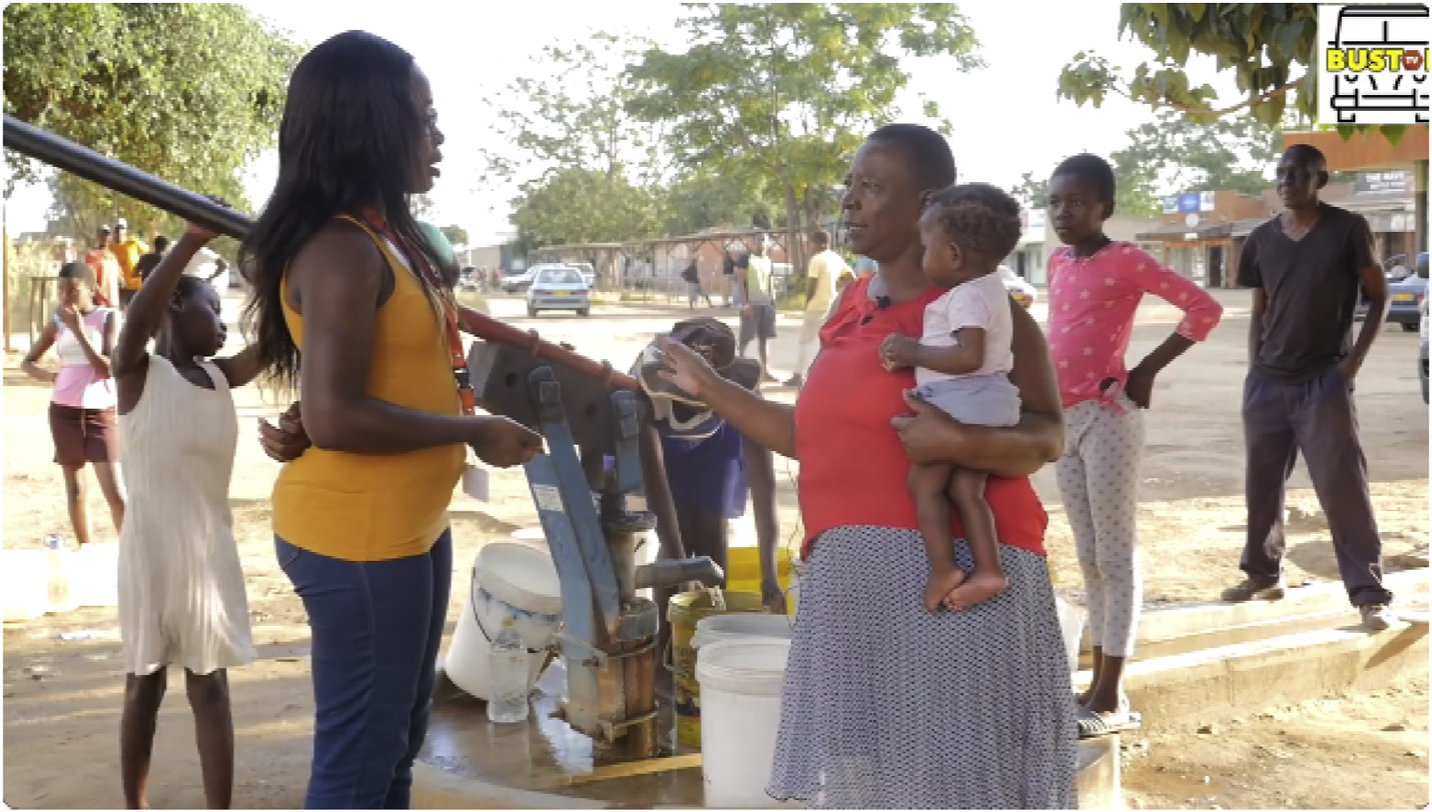
Women and children collecting water at boreholes increasing their risk of contracting COVID-19. **Source:** Bustop TV interview.

Intermittent water supply is becoming more severe in Chitungwiza. The interviewees expressed that since the start of lockdown, their suburbs never had access to potable water. Lack of personal protective equipment, such as masks, was also an issue. At all the boreholes visited, people had no masks despite gathering in large numbers (Fig. 4).

*We heard that in Unit J they had water access for a few hours last Sunday, but here in Unit K we have no water for 12 weeks. We come here with kids. As you can see, I am with my child. (She was breast feeding at the borehole). We do social distancing at home not here. We have no masks; we only heard about them or see them on TVs, but none of us here is wearing a mask as you can see” – Woman at Seke South water point*

In addition to continuous dry taps, other factors such as limited number of boreholes and lack of maintenance also further exposes residents to the risk of contracting COVID-19. This has led to the overutilization of many boreholes causing them to be difficult to operate. This forced many people to touch the force rod. These people were not sanitizing/wash their hands with soap.

*“The hand pump is too heavy; one person cannot operate it. We come here in groups to assist each other to pull the hand pump. So usually 3 or 4 people help each other on the force rod. We touch the force rod without sanitizing. This increases our chances of contracting COVID-19 but we have no option, we have to collect water” – Young women at Seke north borehole.*

Developed countries, having more resources to tackle the disease, highly relied on their water supply system to fight COVID-19. For example, China and other developed countries have been spraying the streets and public areas using chemicals dissolved in water.^6^ While in developing countries like Ethiopia where authorities disinfection of public place led to a disaster of water supply outage.^7^ Thus, revealing that apart from being used for maintaining personal household hygiene, including handwashing, water is the main ingredient in tackling the spread of COVID-19 pandemic in many ways.

Residents of Chitungwiza face much a greater threat from water shortages than those in many other countries where lockdown was imposed. In the event of the pandemic taking its toll in Zimbabwe, Chitungwiza will likely experience extreme casualties due to the lack of access to potable water. To manage COVID-19 lockdowns and water consumption in Chitungwiza. Some Chitungwiza residents have reduced their daily water use as COVID-19 lockdown has reduced access to the water points. Others were fearing that constant fetching of water increases their chances of contracting COVID-19 at boreholes.

*“We have reduced the amount of water we use per day to two 20 L buckets; we fear that coming here frequently increases our risk us of contracting COVID-19.” – Women at Zengeza water.*

**Fig. 5:**
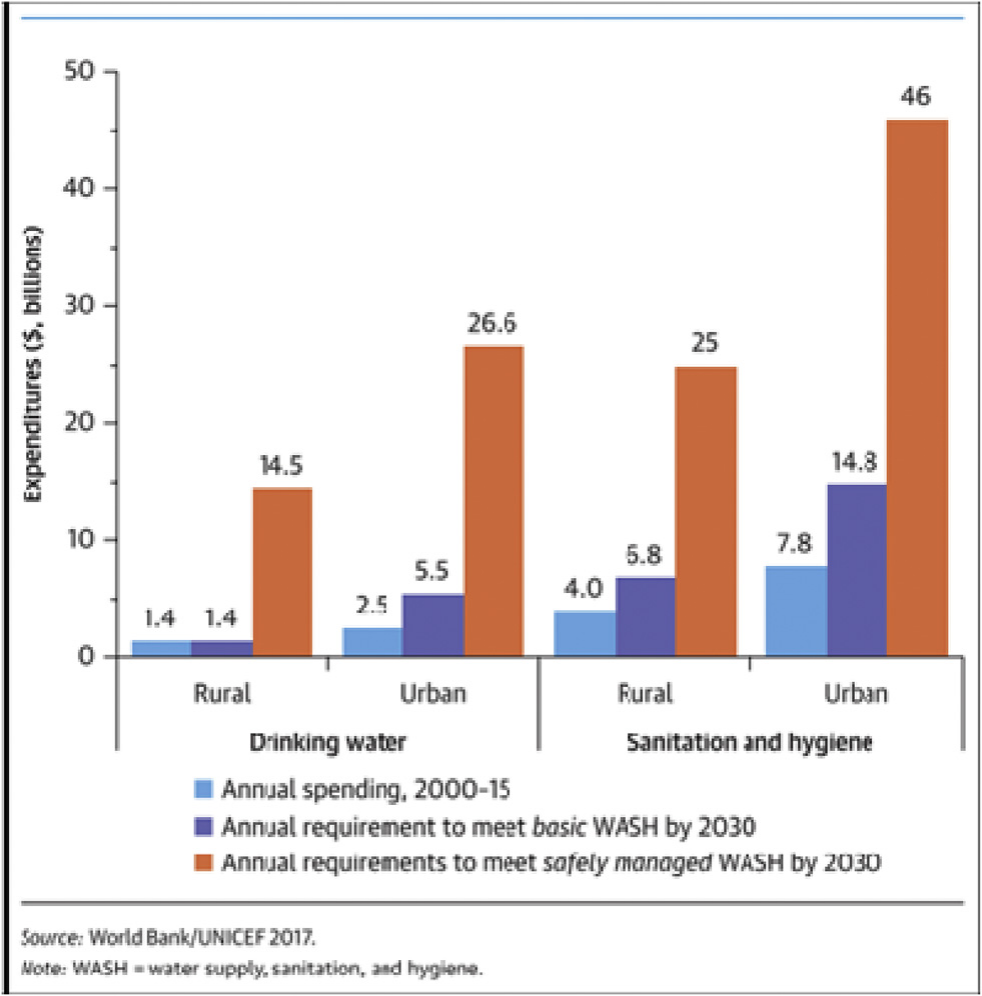
Global investments required to meet SDG 6 (Water for all - safely managed systems) relative to MDGs. **Source:** World Bank Report, 2017: Easing the Transition to Commercial Finance for Sustainable Water and Sanitation.

The unavailability of water in Chitungwiza was not only affecting the WHO ‘Safe Hands’ challenge implementation, but it was also affecting residents in different ways. Residents failed to comply to the recommended hygiene practice, *i.e.* wiping down contaminated surfaces. The lack of water increased the exposure of women and children gathering in long queues at boreholes. This affected the effectiveness of the ‘stay at home’ measures that were implemented by government. Staddon et al. (2020) argued that women and girls as the main domestic water labour in the global South will be more vulnerable to viral infection and intercommunal conflict. They predicted that the current pandemic exacerbates the existing inequalities and vulnerabilities within and between communities. The results in Chitungwiza confirm this as more women and children were engaged in water collection activities during the lockdown. Fig. 5 shows children collecting water at a borehole during the lockdown.

Improving access to water will not act as a standalone solution to curb the spreading of COVID-19 in developing countries. Greater availability and accessibility of other essentials such as soap and sanitizers will also be needed to ensure the success of WHO’s ‘Safe Hands’ challenge in towns and cities in developing countries. Interviewees had no sanitisers or soap.

*“We don’t have sanitisers at home. Shops are running out of sanitizers” – Old Woman at Zengeza water point*

*“Not only that we need water. We don’t have soap at home. Things are expensive in shops” – woman at St Mary’s water point*

These water points are overseen by local management committees. An interview with one of the management committee members of a water point in St Mary’s zone (Mangoromera) explained that the management committee for that water point had set rules aiming to maintain social distancing during COVID-19 to ensure safety of people fetching water. However, there was a lack of enforcement of these rules. These local management committees also do not have the financial resources to ensure that essentials like soaps and sanitizers are available at water points, so that people can wash their hands and disinfect touch points.

*“As you can see, we have a notice here that we erected since the announcement by the President about COVID-19.The notice is showing rules that our people should observe during this pandemic” – Mangoromera water point committee member in St Mary’s zone.*

## 4 What are the possible solutions to water challenges in Chitungwiza?

This article submits possible solutions to manage the tension between stopping the spread of COVID-19 and the need to manage water demand and consumption by Chitungwiza municipality and other municipalities in developing countries to allow a sustainable supply of potable water during COVID-19. To date, it’s unclear when COVID-19 is going to be contained, implying that the WHO ‘Safe Hands’ could be practiced for an indefinite period. The article proposes short-and-long term measures that both municipalities and residents can implement.

Short-term measures are immediate decisions that municipalities and residents can implement to ensure that there is continuous access to water. They aim to limit water use from the residents’ side and increase water supply from the municipalities’ side by strengthening their capacities. Residents in Chitungwiza can limit activities that consume water such as watering their gardens in order to prioritise health promoting activities, including the regularly washing hands. Changes of behaviour regarding water use reduce the daily water use (Sorensen, 2017), and allocate the available water for washing hands and surfaces. Prioritization of water use at the household level has been used in several cases during periods of droughts with positive results. Research in Cape Town (Sorensen, 2017) has shown that residents are capable of making unprecedented behavioral and attitudinal shifts and technological innovation (Simpson et al., 2019b), to better manage water during stringent household water supply.

Increasing residents’ social awareness of household-level water management practices, such as the opening and closing of taps when rinsing hands, can significantly reduce consumption during this pandemic time and beyond. There are numerous examples demonstrating the impact of social awareness campaigns in reducing water consumption. When Cape Town, South Africa experienced a 3-year drought, a substantial media campaign was launched to inform residents about the severity of the drought and to urge relentless water conservation (Booysen et al., 2019). In response, residents reduced water usage from 540 to 280 L per household per day between January 2015 and January 2018 (Booysen et al., 2019). Together with higher water tariffs, this communication campaign was credited for playing an important role in shifting water consumption patterns and related attitudes (Booysen et al., 2019). This demonstrates the significant role social awareness plays in the reduction of water usage. Chitungwiza and other under-resourced municipalities can implement these measures during the COVID-19 pandemic to allow a more constant supply.

In addition to the above, this article presents other key measures that households within urban areas of Africa can implement. Table 2 summarises these measures.

**Table 2:** Key water management measures that water stressed regions can implement to maintain ‘Safe Hands’ during COVID-19.

Source	Measures
WHO (2020)	- Wash your hands with soap and running water when your hands are visibly dirty, if they are not visibly dirty, use alcohol-based hand rub (ABHR) frequently.
Haddout et al. (2020)	- Don’t let the tap or shower run when it is not being used. - Run the dishwasher and clothes washer only when they are fully loaded. - Fix leaks to conserve water and also save on repair costs. - Education (public awareness). - Setting up awareness programs through social media for sharing information and for providing advice, training and capacity building support. - Allocate adequate amount of water for drinking and hand washing in each community at important locations where public gathering and crowds are noticed. - Ensure alternatives to hand washing are available such as the use of hand sanitizers, in the case of extreme lack of water resources.

However, the household roles above cannot replace the responsibility of municipalities to ensure reliable water supply services to the people. Haddout et al. (2020) also emphasised the importance of municipalities in developing countries to provide running water for the effectiveness of regular handwashing to stop COVID-19 transmission. The above household measures are rudimentary; they work as complementary measures to the municipality providing running water to households 24/7. The municipalities ability to provide such continuous services can be strengthened in several ways. One way is through the central governments urgently exhausting various financial schemes such as grants to fund water supply projects. This gives assurance of running water on household taps in this critical time and for the ‘Safe Hands’ challenge to yield positive results in cities of low-income countries. This underlines the importance of budgetary flexibility, robust financial buffers and insurance mechanisms for cities in developing countries.

The current water supply situation in Chitungwiza reflects how lack of inter- and intra-governmental coordination within and between central and local governments across sectors can exacerbate inefficiencies and delay response. This concurs with Nyikadzino and Nhema (2015) findings that Chitungwiza’s poor water supply services are attributed to the lack of coordination between the council and the central government. Immediate government intervention is required to address water issues in Chitungwiza to protect thousands from the COVID-19.

Private sector elsewhere has been a key stakeholder in the provision of water supply mostly in developed regions and the global west (Dahiya and Gentry, 2020; Fu and Guo, 2020; Purbo et al., 2019; Ruiters and Matji, 2016). On securing a sustainable water supply service, Chitungwiza can sign concessions with the private sector forming public private partnerships (PPPs). These concessions are crucial to encourage huge investments needed to expand and improve water infrastructure supply and related services. These long-term planning investments in water, like elsewhere in developed regions, improves water services efficacy (Arezki, 2020; Ruiters and Matji, 2016; Dahiya and Gentry, 2020; Purbo et al., 2019; Qian et al., 2019). Further research is required on how these mechanisms can work in the context of Chitungwiza.

The global call for ‘Safe Hands’ has exposed how weak the water governance is in developing regions. Long-term measures to address the lack of access to potable water by many urban settlements require structural reforms of municipalities and other relevant institutions. In many developing countries, institutions responsible for water service provision are still trying to solve new problems with old solutions (Simpson et al., 2019a). Experience in such circumstances shows little improvements compared to the investments committed (Dasgupta, 2019). Water utilities and municipalities in developing regions are still heavily investing in linear systems, “big pipes in and big pipes out” transfer models (Aboelnga et al., 2020; Tjandraatmadja, 2019). The soft path approach that focuses on strengthening integrated urban water supply governance has been inadequately addressed in many countries. Institutions continue to work in silos - policies are set without aligning objectives with the required resources and are counting on public funds that are insufficient and poorly targeted (Aboelnga et al., 2020). The new sources of finance for water supply in urban areas in developing countries is constrained by regulatory, institutional and other barriers. New approaches to address the water governance in developing countries are required.

To demonstrate the importance of flexible budgetary planning for water management during critical times such as the COVID-19 pandemic, we assessed the interventions done by the Department of Water and Sanitation in South Africa (Box 4) to compare with the situation in Zimbabwe.

Box 4 **COVID-19 and Safe Hands response by Department of Water and Sanitation in South Africa.**

To track the progress of the ‘Safe Hands’ challenge in the region, South Africa was used as an example focusing on its response to the WHO ‘Safe Hands’ approach in the fight against the COVID-19. Responding to COVID-19 outbreak and WHO challenge, South Africa, through the Department of Water and Sanitation proposed a series of interventions around human settlements, water and sanitation. They pledged to; prioritise the maintenance of water infrastructure to allow continuous supply of water to settlements (this is positive for ‘Safe Hands’ challenge) focusing on areas with existing water challenges and those frequently experiencing droughts. The department prioritised high risk areas including public areas with limited or no access to water, overcrowded and informal settlements and water scarce towns. When the time they published their statement of intent; the Department had identified 2000 communities that needed urgent attention, making South Africa one of the quickest countries to respond to the ‘Safe Hands’ challenge. Their priority list had communities not yet served by a formal water service connection which is usually informal settlements in developing countries. The intervention included providing communal water storage with water collection points filled by water carting managed by the Department of Water and Sanitation. To fast track the implementation of these interventions, the Department set up an Operation Centre. The setting up of the centre shows a huge commitment to ensure successful implementation of the ‘Safe Hands’ challenge. *(Source: Department of Water and Sanitation, Republic of South Africa. Press Statement on March 24, 2020)*.

The case of South Africa does not reflect only the importance of investing in water infrastructure but, also the importance of coordinated planning in water governance during periods of crisis. Despite South Africa and Zimbabwe sharing a border, the two countries represent two different scenarios. The South African national government and municipalities learned from the lessons of the preceding 2015–2018 drought. The drought in Cape Town, South Africa stressed the importance of inter-governmental cooperation and coordinated planning by several stakeholders. The drought required inter- and intra-governmental cooperation between multiple spheres of government, including the management of a broad range of stakeholders and political entities (Nhamo and Agyepong, 2019). The Cape Town drought highlights how a lack of coordination between essential organs of state and political entities can exacerbate and slow response efficacy (Rodina, 2019). African cities can learn from Cape Town how improved planning for more resilient systems through building viable partnerships and communication channels can enhance coordination and cooperation between local, regional and national levels of government (Parks, 2019).

Alt-text: Box 4

Financial mechanisms are required to finance the long-term investments needed in developing regions to improve water access. Investments, however, should not just prioritise infrastructure development because experience has shown that infrastructure investments alone do not give the much-needed change in the water sector. Investments in governance systems, planning and implementation are also vital. Transformational and structural reforms of these municipalities is vital to address the water problems in developing regions. Policies that allow sustainable water resources development and their implementation are also vital in regions lacking water access (Haddout et al., 2020). In order to effectively change the status quo, decision makers and municipalities require conceptual and operational shifts in ways of doing things (Simpson et al., 2019b), including but not limited to, inter-governmental cooperation and management (Rodina, 2019), financial planning that anticipates disruptive shocks to municipal budgets (Simpson et al., 2019c), alternative supplies development (Olivier and Xu, 2019) and enhanced crisis communication (Booysen et al., 2019).

Water points/boreholes have emerged as new sources of water in response to chronic intermittent water supplies in Chitungwiza. However, their existence is insufficient as demonstrated during interviews where more than 300 people were gathering at one borehole. This increases their chances of contracting and spreading COVID-19, defeating the purpose of the remedial ‘stay at home’ orders that were implemented worldwide and in Zimbabwe to limit public gatherings. One solution could include constructing more boreholes to improve water access in the suburbs of Chitungwiza, as to limit the number of people gathering at and using one borehole. Effective management of the existing boreholes is also important as some of the boreholes in the study area are not working.

The outbreak of COVID-19 should be a wakeup call to developing countries on the changes needed to achieve Sustainable Development Goal (SDG) 6. Future pandemics may continue to disrupt the achievement of the SDGs specifically around water for all. This should serve as an opportunity for governments and local authorities to foster cooperation and bilateral agreements that improve water access. With the establishment of ambitious SDG 6 in 2015, governments set the bar higher than ever before for improving water access, shifting from basic to safely managed systems, and well as calling for universal access to these essential services (Aboelnga et al., 2020). Safely managed systems are critical during period of uncertainty associated with climatic- and health-related hazards such as droughts and COVID-19. However, achieving safely managed systems would remain a far-fetched idea in developing countries as huge political and financial commitments are required. Fig. 5 shows the global investments required to meet SDG 6 by 2030 compared with the annual spending recorded for achievement of the Millennium Development Goals (MDGs) (2000–15).

Fig. 5 shows a wide gap between previous MDG spending and what is needed to achieve SDG 6 to ensure safely managed water systems for all. Most of these countries with people lacking water access are in developing regions that lack the financial mechanisms to invest in water systems. Conditions such as COVID-19 present further unforeseen challenges that slow the progress on water investments. This is because governments and municipalities shift their focus to ‘firefighting’, emergency approaches regardless of the fact that long-term reliable water access is the key weapon in this and future fights. For developing countries that lack investments and financial mechanisms to meet the targets laid out by SDG 6 (Fig. 5), strategic planning is required. Events such as COVID-19 should be utilised as opportunities to improve cooperation and bring the world’s attention to one goal: improved water access for all.

The outbreak of COVID-19 has revealed that a single event can prompt previously unseen human and economic losses at a large scale (Aboelnga et al., 2020) and how conditions such as the lack of water may accelerate these losses. A new paradigm is needed that turns on its head the current approach to water development and the ways in which institutions and governments in developing regions manage and finance urban water security (Aboelnga et al., 2020). Far-sighted and highly capacitated water planning and governance have been identified as important in addressing water supply challenges in Africa and elsewhere (Muller, 2018).

## 6 Conclusions and recommendations

The COVID-19 outbreak brings up the vital importance of improved water access in developing countries, particularly in poor, informal and high-density urban areas. This pandemic is a wake-up call to the importance of achieving SDG 6. Vulnerability to COVID-19 for people in developing countries is increased by factors such as the lack of household water access and the unavailability of soap, alcohol based hand rubs and disinfectants. They are the main barriers to the implementation and efficacy of the WHO ‘Safe Hands’ initiative in Chitungwiza. Water scarcity does not only affect the ‘Safe Hands’ initiative, but also the effectiveness of national lockdowns in developing areas. In Chitungwiza, women and children, given their exposure when collecting water without adequate protection, are at higher risk from COVID-19. These public places often pull together crowds that have no access to sanitizers nor soap. Dry household taps only give one alternative to inhabitants of Chitungwiza: ignore social distancing measures to combat coronavirus, so that they may access shared waterpoints to collect water for drinking and hygiene purposes. The study further illustrates how the lack of reliable water supplies for cities in developing countries decreases people’s resilience towards diseases and pandemics. While residents in Chitungwiza clearly understood the importance of regular handwashing, the lack of access to water barred them from practicing the required hygienic activities. The increase in water demand due to regular handwashing has compounded the effects in the under-resourced municipalities. The existing multiple stressors in these regions aggravate the situation.

As short-term measures, we recommend households take the lead in practicing domestic water management. We recommend temporary changes to behaviours or the adoption of new water use regimes. This can be reinforced through extensive social awareness campaigns directed to residents to conserve water, such as encouraging and educating residents to practice measures such as opening and closing faucet/taps when rinsing hands. Residents can also suspend water consuming activities such gardening to prioritise the available water for washing hands.

However, the household role above cannot replace the responsibility of municipalities to ensure reliable water supply services to people. These municipalities need capacitation. Central governments in developing countries should also consider immediate financial assistance to the under-resourced municipalities through grants. Municipalities on can also partner with the private sector to form public private partnerships (PPPs) to fund both immediate and long-term water investments. Municipalities should implement structural and transformational reforms that enhance investments both around water infrastructure and governance to allow flexible planning. This can cushion residents against future pandemic outbreaks.

## Funding

The research did not receive any financial grant.

## Declaration of competing interest

The authors declare that they have no known competing financial interests or personal relationships that could have appeared to influence the work reported in this paper.

## Footnotes

1 WHO save lives: clean your hands in the context of COVID-19. A handout with key guidelines on the importance of safe hands initiative and how it can be done. Found on https://www.who.int/docs/default-source/coronaviruse/who-hh-community-campaign-finalv3.pdf?sfvrsn=5f3731ef_2.

2 https://www.afdb.org/en/news-and-events/coronavirus-and-climate-change-observing-world-water-day-34912.

3 https://unstats.un.org/sdgs/report/2019/goal-11/.

4 A site shows the latest population census for cities across the globe. We used the site statistics because the last census in Zimbabwe was done in 2012 (8 years ago), next census is 2022. Found on https://populationstat.com/zimbabwe/chitungwiza.

5 Four zones are Seke North and South, Zengeza and St Mary’s as adopted in the Chitungwiza Water Department SLB Report of 2016.

6 https://www.businessinsider.com/wuhan-coronavirus-china-dispatches-bleach-trucks-to-spray-down-cities-2020-2.

7 https://www.ethiopiaobserver.com/2020/05/31/the-pandemic-is-laying-bare-a-global-water-crisis/.
